# Brazilein from *Caesalpinia sappan* L. Antioxidant Inhibits Adipocyte Differentiation and Induces Apoptosis through Caspase-3 Activity and Anthelmintic Activities against *Hymenolepis nana* and *Anisakis simplex*

**DOI:** 10.1155/2013/864892

**Published:** 2013-03-11

**Authors:** Chia-Hua Liang, Leong-Perng Chan, Tzung-Han Chou, Feng-Yu Chiang, Chuan-Min Yen, Pin-Ju Chen, Hsiou-Yu Ding, Rong-Jyh Lin

**Affiliations:** ^1^Department of Cosmetic Science, Chia Nan University of Pharmacy and Science, Tainan, Taiwan; ^2^Institute of Clinical Medicine, Kaohsiung Medical University, Kaohsiung, Taiwan; ^3^Department of Otolaryngology-Head and Neck Surgery, Kaohsiung Medical University Hospital, Kaohsiung Medical University, Kaohsiung, Taiwan; ^4^Department of Chemical and Materials Engineering, National Yunlin University of Science and Technology, Yunlin, Taiwan; ^5^Department of Parasitology, Faculty of Medicine, Kaohsiung Medical University, Kaohsiung, Taiwan; ^6^Institute of Cosmetic Science, Chia Nan University of Pharmacy and Science, Tainan, Taiwan

## Abstract

Brazilein, a natural, biologically active compound from *Caesalpinia sappan* L., has been shown to exhibit anti-inflammatory and antioxidant properties and to inhibit the growth of several cancer cells. This study verifies the antioxidant and antitumor characteristics of brazilein in skin cancer cells and is the first time to elucidate the inhibition mechanism of adipocyte differentiation, cestocidal activities against *Hymenolepis nana*, and reduction of spontaneous movement in *Anisakis simplex*. Brazilein exhibits an antioxidant capacity as well as the ability to scavenge DPPH^•^ and ABTS^•+^ free radicals and to inhibit lipid peroxidation. Brazilein inhibited intracellular lipid accumulation during adipocyte differentiation in 3T3-L1 cells and suppressed the induction of peroxisome proliferator-activated receptor **γ** (PPAR**γ**), the master regulator of adipogenesis, suggesting that brazilein presents the antiobesity effects. The toxic effects of brazilein were evaluated in terms of cell viability, induction of apoptosis, and the activity of caspase-3 in BCC cells. The inhibition of the growth of skin cancer cells (A431, BCC, and SCC25) by brazilein is greater than that of human skin malignant melanoma (A375) cells, mouse leukemic monocyte macrophage (RAW 264.7 cells), and noncancerous cells (HaCaT and BNLCL2 cells). The anthelmintic activities of brazilein against *Hymenolepis nana* are better than those of *Anisakis simplex*.

## 1. Introduction

All biological systems that operate under aerobic conditions are exposed to oxidants that are generated either intentionally or as byproducts [1]. Multiple species of oxidants are being generated, as implied by the first letters of the terms “reactive oxygen species” (ROS) and “reactive nitrogen species” (RNS) [2]. When present in foods or body at lower concentrations than that of an oxidizable substrate, antioxidants considerably delay or prevent the oxidation of that substrate. Antioxidants may help the body to protect itself against various forms of oxidative damage that are caused by ROS and RNS, which are associated with various diseases including cancer, diabetes, shock, arthritis, and acceleration of the ageing process [3]. 

Reduced antioxidant capacity, the increased production of ROS, and the elevated oxidation products of lipids, DNA, and proteins have been identified in plasma, urine, and various tissues suggesting systemic and organ-specific oxidative stress [4]. Recent methods of systemic oxidative stress include the detection of increased circulating and urinary levels of the lipid peroxidation product F2-isoprostane in both type I and type II diabetic patients and in obese persons [5]. Obesity is a serious public health problem because of its association with the development of various diseases, including diabetes, cardiovascular disease, osteoarthritis, and many forms of cancer [6]. Adipogenesis which plays an important role in obesity involves the development of preadipocytes to mature adipocytes with the accumulation of lipid droplets, increased fat cell size (hypertrophy), and an increase in the number of cells or hyperplasia [7]. Therefore, the reduction of obesity may involve the inhibition of the adipogenic process and lipid accumulation owing to dedifferentiation, lipolysis, and apoptosis [8]. 

*Hymenolepis nana* is the most common cause of all cestode infections and is found globally. In temperate zones, its incidence is high in children and institutionalized groups.*H. nana* infections are typically asymptomatic, but heavy infections by *H. nana* can cause headaches, weakness, anorexia, abdominal pain, and diarrhea [9]. *H. nana *is the only cestode capable of completing its cycle without an intermediate host [10]. Infection is most commonly acquired from eggs in the feces of another infected individual, which are transferred by contamination in food. Eggs hatch in the duodenum, releasing oncospheres, which penetrate the mucosa and come to lie in the lymph channels of the villi. The oncospheres develop into a cysticercoid, which has a tail and a well-formed scolex. It is made of longitudinal fibers and is spade-shaped with the rest of the worm still inside the cyst. In five to six days cysticercoids emerge into the lumen of the small intestine, to which they attach before they mature. Eggs of *H. nana* are immediately infective when passed with the stool and transferred in contaminated food. Eggs are ingested by an arthropod intermediate host and hatch in the duodenum, releasing oncospheres (hexacanth larvae), which penetrate the intestinal villus and develop into cysticercoid larvae, which have a tail and a well-formed scolex. Upon rupture of the villus, the cysticercoids return to the intestinal lumen, evaginate their scoleces, attach to the intestinal mucosa and mature into adults that reside in the ileal portion of the small intestine, producing gravid proglottids. The eggs are then passed in the stool when released from the proglottids through their genital atrium or when the proglottids disintegrate in the small intestine. An alternate mode of infection consists of internal autoinfection, in which the eggs release their hexacanth embryo, which penetrates the villus, continuing the infective cycle without passing through the external environment.The short life span and rapid course of development also facilitate the spread and ready availability of this worm, but internal autoinfection allows the infection to persist for years [10, 11]. 

*Anisakis simplex* adult worms in the primary host gastric mucosa release eggs that are passed in the feces; in seawater, the eggs are embryonated and form first-stage (AsL1) and subsequently free-living second-stage larvae (AsL2). When ingested by small crustacean first-intermediate hosts (such as krills), the parasite matures into *A. simplex* third-stage larvae (AsL3) that are subsequently consumed by marine fish or squid (second intermediate hosts) (such as codfish, hake, mackerel, cuttlefish, and sardines). The AsL3 larvae migrate into the viscera and peritoneal cavity; the degree of migration into the fish musculature may depend on environmental conditions and/or the species of parasite and fish [12, 13]. They are often transferred among fish along the food chain, and consequently, piscivorous fish may accumulate large numbers of larvae [13]. Ultimately, the ingestion of infected fish or squid by a marine mammal (final host) leads to the development of fourth-stage larvae and then adults. Humans can become accidental hosts by consuming undercooked and/or raw second-intermediate hosts that contain the AsL3. These parasites rarely develop further within the human gastrointestinal tract, instead, by means of proteolytic enzymes, but they typically become embedded in the gastric or intestinal mucosa and die or invade the muscular layers of the stomach and intestine to induce allergic reactions and a variety of abdominal symptoms, which are characterized as anisakiasis or anisakidosis [14]. The four main clinical syndromes in humans who experience symptomatic anisakidosis include gastric, intestinal, ectopic (or extra-gastrointestinal), and allergic diseases.

Anisakidosis is globally recognized as a public health hazard. It is relatively common in Asia and Europe [15, 16]. The prevalence of the disease has increased markedly because of the increasing popularity of Japanese cuisine, such as “sushi” and “sashimi.” The pathology of Anisakis is attributable to two main mechanisms-allergic reactions and direct tissue damage [17], which results in violent abdominal pain, nausea, and vomiting, which symptoms mimick gastric ulcers and appendicitis. Moreover, the penetration of *A. simplex* into the stomach and bowel wall produces a severe eosinophilic granulomatous response. Although anisakiasis generally evolves toward spontaneous resolution, some patients will develop serious complications such as obstruction, perforation, and peritonitis [18], which sometimes require surgery [13]. The availability of an anthelmintic compound against Anisakis larvae has the potential to shorten the clinical course and prevent mechanical complications that arise from endoscopic procedures. The effectiveness of treatment with anthelmintic agents, antibiotics, anticholinergics, and/or corticosteroids against *A. simplex* remains controversial, and few studies have shown the effectiveness of anthelmintic drugs against *A. simplex *[19].

*Caesalpinia sappan *L. is a traditional medicine plant that is produced in Taiwan, China, India, Myanmar, Vietnam, Sri Lanka, and Malay Peninsula [20]. The dried heartwood of *C. sappan* L. has been used for a long time in oriental folk medicines. It is traditionally applied as an aqueous decoction and prescribed to invigorate the blood system, promote menstruation, and reduce pain and swelling [21]. Additionally, *C. sappan* L. have been recommended for medicinal purposes owing to its astringent or diuretic properties, as well as for treating certain skin diseases [22]. *C. sappan* L. extracts have been shown to exhibit anti-inflammatory, antioxidative, antimicrobial, antiviral, antitumor, antiatherosclerosis, hypoglycemic, and spasmolytic activities and to promote blood flow [20]. Brazilein, the unique tetracyclic homoisoflavonoid from *C. sappan* L., is a natural red pigment that is utilized in traditional Chinese medicine [23]. Previous investigations have demonstrated that brazilein exhibits a positive inotropic action in isolated cardiac tissues and inhibits Na^+^, K^+^-ATPase activity, anti-complementary activity on the immune system, and the scavenging of hydroxyl radicals [23, 24]. In lipopolysaccharide-(LPS-)stimulated microglial BV2 cells, brazilein suppresses the release of nitric oxide (NO), tumor necrosis factor-*α* (TNF-*α*), and interleukin (IL)-6 and reduces the expression of inducible nitric oxide (iNOS) synthase, which may protect the brain against ischemia/reperfusion injury [25]. Brazilein partially inhibits rat aorta contractions that are induced by ergometrine maleate (5-hydroxytryptamine 2 receptor agonist) [26]. Moreover, brazilein exhibits significant cytotoxic activity against HepG2 and Hep3B (liver), MDA-MB-231 and MCF-7 (breast), A549 (lung), and Ca9-22 (gingival) human cancer cell lines [27]. Nevertheless, current investigations of the anti-skin cancer properties of brazilein and its larvicidal effects on *H. nana* and *A. simplex *are insufficient. This study confirms the antioxidant and antitumor effects of brazilein and elucidates novel mechanisms for its inhibition of adipocyte differentiation, its larvicidal activity against *H. nana, *and its elimination of the spontaneous mobility of *A. simplex*.

## 2. Materials and Methods

### 2.1. Reagents and Cell Lines 

1,1-Diphenyl-2-picrylhydrazyl (DPPH^•^), 2,2′-azinobis(3-ethylbenzothiazoline-6-sulfonic acid) diammonium salt (ABTS^•+^), 2,5,7,8-tetramethylchroman carboxylic acid (trolox), and 3-isobutyl-1-methylxanthine (IBMX) were purchased form Sigma Chemical Co. (St. Louis, USA). 3T3-L1 preadipocytes, human epidermoid carcinoma A431, human oral squamous cell carcinoma SCC25, human skin malignant melanoma A375, mouse leukemic monocyte macrophage RAW 264.7, and mouse normal embryonic liver BNLCL2 cells were purchased from the American Type Culture Collection (Rockville, MD, USA). Human basal cell carcinoma BCC and human premalignant keratinocytic HaCaT cells were kindly provided by Professor Hamm-Ming Sheu (National Cheng Kung University Medical College, Tainan, Taiwan). Cells were cultured in medium supplemented with 10% fetal bovine serum (Hazelton Product, Denver, PA, USA) and 1% penicillin-streptomycin at 37°C in 5% CO_2_ humidified atmosphere; specifically, SCC25 were maintained in DMEM/F12 medium (GIBCO, Grand Island, NY, USA) supplemented with 0.4 **μ**g/mL hydrocortisone (Sigma, St. Louis, MO, USA), 3T3-L1, A431, A375, HaCaT, RAW 264.7 and BNLCL2 cells in DMEM, and BCC cells in RPMI medium. 

### 2.2. Extraction and Isolation of Brazilein 

The dry powder of *C. sappan* heartwood (33.0 kg) was extracted with 95% ethanol at room temperature. After removal of the solvent by evaporation, the residue (3.45 kg) was partitioned with water and ethyl acetate (1 : 2). The ethyl acetate layer was removed by evaporation and the residue was then suspended in methanol-water (9.5 : 0.5) and partitioned with *n*-hexane (1 : 1). The methanol layer was subjected to silica gel column chromatography and eluted with *n*-hexane-ethyl acetate (7.5 : 2.5, 1 : 1, 2.5 : 7.5), ethyl acetate, ethyl acetate-methanol (1 : 1), and methanol, successively. Each fraction collected from the column was monitored by thin-layer chromatography and the similar fractions were combined to produce 8 fractions. The fraction 4 was further purified by silica gel column and eluted with *n*-hexane-ethyl acetate (7.5 : 2.5, 1 : 1, 2.5 : 7.5) and ethyl acetate to isolate brazilein (127.5 g). Their structures were confirmed by NMR and mass spectra analysis.

**Figure 1:**
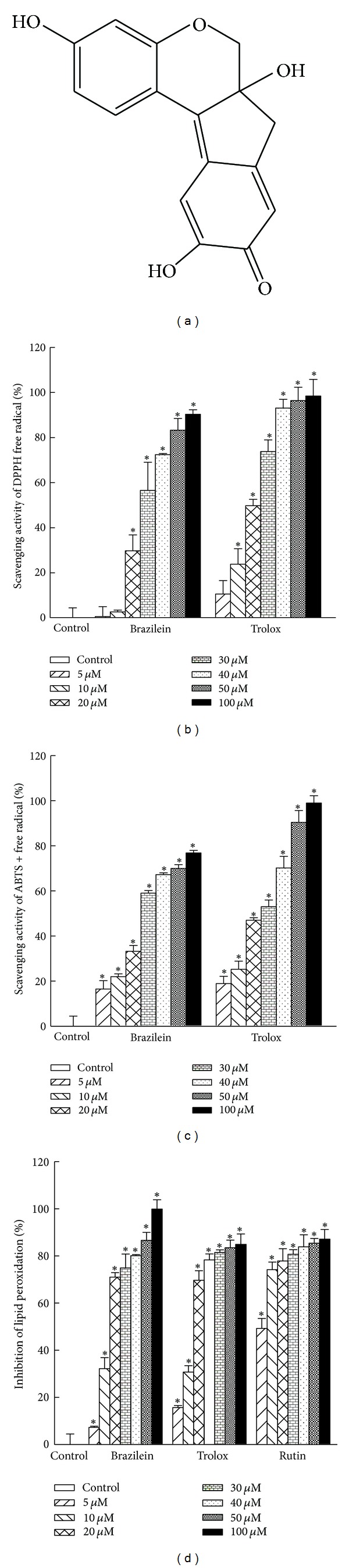
Antioxidant activity of brazilein. (a) Chemical structure of brazilein from *C. sappatin *L. M.W. = 284. (b) DPPH^•^ and (c) ABTS^•+^ free radical scavenging activities of brazilein and trolox (5, 10, 20, 30, 40, 50, and 100 **μ**M). (d) Inhibition of lipid peroxidation by brazilein, trolox, and rutin (5, 10, 20, 30, 40, 50, and 100 **μ**M) using liposome as an oxidizable substrate. Data are presented as mean ± SD from three independent experiments; *P < 0.05 indicates significant difference from vehicle-treated cells.

Brazilein: reddish-brown powder; m.p. 261–264°C; ESI/MS *m/z*: 285 [M-H]^+^; ^1^H-NMR (pyridine-d_5_, 500 MHz) **δ**: 2.91 (1H, d, *J *= 17.5 Hz, H-7a), 3.24 (1H, d, *J *= 17.5 Hz, H-7b), 4.12 (1H, d, *J *= 11.5 Hz, H-6a), 4.84 (1H, d, *J *= 11.5 Hz, H-6b), 6.66 (1H, s, H-8), 6.87 (1H, d, *J *= 2.0 Hz, H-4), 6.92 (1H, dd, *J *= 8.8, 2.0 Hz, H-2), 7.59 (1H, s, H-11), 8.00 (1H, d, *J *= 8.8 Hz, H-1); ^13^C-NMR (pyridine-d_5_,125 MHz) **δ**: 181.5 (C-9, C=O), 164.7 (C-3), 160.0 (C-7a), 159.6 (C-4a), 154.9 (C-10), 152.7 (C-12), 131.8 (C-1), 124.6 (C-11a), 119.3 (C-8), 112.6 (C-1a), 112.6 (C-2), 105.7 (C-11), 104.9 (C-4), 76.0 (C-6a), 74.7 (C-6), 41.5 (C-7). These data were compared with literature values [28]. The chemical of brazilein was shown in Figure 1(a). The purity of brazilein is 99.3%. The solubility of brazilein was 100 mM in DMSO.

### 2.3. Radical Scavenging Ability against DPPH^•^ and ABTS^•+^ of Brazilein 

The free radical scavenging activities of brazilein were determined by DPPH^•^ and ABTS^•+^ scavenging assays as previously reported [29]. Briefly, 5, 10, 20, 30, 40, 50, and 100 **μ**M brazilein (10 **μ**L of solution) was mixed with 90 **μ**L of dimethylsulphoxide (DMSO) and 900 **μ**L of ethanolic DPPH^•^ solution (0.1 mM). After incubation in darkness at 25°C for 30 min, the absorbance (*A*) was determined at 517 nm (Hitachi U-2001, Japan). The inhibition percentage of ABTS^•+^ radical as determined by the extent of decolorisation was taken to be proportional to the concentration of antioxidants. ABTS^•+^ was dissolve in water to a 7 mM concentration. ABTS^•+^ radical was produced by reacting ABTS^•+^ stock solution with 2.45 mM potassium persulfate and the mixture to stand in the dark at room temperature for 12–16 h. The ABTS^•+^ radical solution was diluted to an absorbance of 0.70 ± 0.02 at 734 nm at 30°C. Each agent (0.1 mL) was reacted with 2.9 mL of diluted ABTS^•+^ radical solution for 20 min at 30°C, and then the absorbance was measured at 734 nm (Hitachi U-2001, Japan). TEAC (trolox equivalent antioxidant capacity) is calculated by comparison with the reactivity of the standard, trolox. Ethanol or distilled water was used as negative controls while trolox served as the positive control. The ability of brazilein or trolox to DPPH^•^ and ABTS^•+^ scavenging activities was calculated using the following equation: radical scavenging ability (%) = (1 − *A*_sample_/*A*_control_) × 100. Percentage scavenging activity was calculated, while EC_50_ values were estimated from the percent inhibition versus concentration plot. The data are presented as mean values ± standard  deviation (*n* = 3). 

### 2.4. Inhibition of Lipid Peroxidation of Brazilein 

The inhibition of lipid peroxidation by brazilein (5, 10, 20, 30, 40, 50, and 100 **μ**M) was determined by using the liposome as a model of biological membranes, as described previously [30]. Fe^2+^/ascorbate-induced lipid peroxidation was used. The liposomes were obtained by dispersing lipids in demineralized water (1 : 10). For the assay, 32 **μ**L of suspension of liposomes was incubated together with 11 **μ**L of 10 mM FeSO_4_, 11 **μ**L of 10 mM ascorbic acid, and appropriate amounts of different concentrations (ranging from 0–100 **μ**L/mL of stock solution) of brazilein, trolox, and rutin in 1.515 mL of 50 mM Na_2_HPO_4_-NaH_2_PO_4_ buffer, pH 7.4 (2.5 ml final solution) at 37°C for 1 h. Lipid peroxidation was terminated by the reaction of 0.8 mL of 1% 2-thiobarbituric acid and 10% trichloroacetic acid and 106 **μ**L of 0.1 M ethylene diamine-tetraacetic acid disodium salt dehydrate at 100°C for 20 min. After cooling and centrifugation (2600 g for 10 min), the malonaldehyde content was determined by measuring the absorbance (*A*) at 532 nm. A control with DMSO instead of sample was also analyzed and expressed no activity. Trolox and rutin were utilized as standards. The inhibition of lipid peroxidation (%) = (1 − *A*_sample_/*A*_control_) × 100. Percentage inhibition was calculated, while EC_50_ values were estimated from the percent inhibition versus concentration plot. The data are presented as mean  values ± standard deviation (*n* = 3).

### 2.5. Cell Viability 

Cells (1 × 10^5^  cells/mL) were seeded in 100 **μ**L of 96-well plates and incubated for 24 h to allow the cells to attach, before treatment with brazilein. Brazilein was dissolved in DMSO and made up with the medium so that the final concentration of the vehicle was not >0.1% DMSO. The cells were treated with brazilein (5, 10, 20, 30, 40, and 50 **μ**M) for 72 h. Cell viability was determined by using the 3-(4,5-dimethylthiazol-2-yl)-2,5-diphenyltetrazolium bromide MTT assay as reported [31]. Absorbance values were expressed as a percentage of vehicle controls (0.1% DMSO), and agents' concentrations resulting in cell growth inhibition of 50% (IC_50_) were calculated.

### 2.6. Adipocyte Differentiation, Treatment, and Oil Red O Staining

Cultivation of 3T3-L1 cells and their conversion to adipocytes were carried out according to the method as described previously [32]. To induce differentiation, four-day postconfluent 3T3-L1 preadipocytes were stimulated for 72 h with 10% FBS/DMEM containing the MDI hormone mixture (0.5 mM IBMX, 1 **μ**M dexamethasone, and 10 **μ**g/mL of insulin) in six-well plates. On day four, the medium was replaced with 10% FBS/DMEM medium containing 10 **μ**g/mL of insulin. The medium was replaced with fresh medium (10% FBS/DMEM, 10 **μ**g/mL of insulin) every two days until analysis on day eight. Brazilein (5 **μ**M) was added during the differentiation process. 

Differentiated 3T3-L1 cells were stained using the Oil Red O method [33]. At day eight of differentiation, the cells were washed with PBS and fixed with 10% formaldehyde for 2 h. The fixed cells were washed with 60% isopropanol and stained with 0.2% Oil Red O for 10 min. The plates were rinsed three times with water and examined under a phase contrast inverted light microscope (Nikon, TE2000-U, Japan). After through washings with water and evaporation of excess water, Oil Red O was extracted in isopropyl alcohol and the absorbance was monitored at 520 nm (BioTek, Synergy 2). 

### 2.7. RNA Isolation and Reverse Transcription-Polymerase Chain Reaction (RT-PCR) Analysis 

3T3-L1 cells were treated with vehicle control (DMSO) or brazilein (5 **μ**M) during the differentiation process. Total RNA was isolated from cells using the Trizol reagent (Invitrogen, Carlsbad, CA, USA), and a RT-PCR was conducted using 3 **μ**g of total RNA and the Superscript cDNA Preamplification System (Weiterstadt, Germany) according to the manufactures' instructions. The following primers were utilized: right primer 5′-GCT CTA GAC GTG ACA ATC TGT CTG AGG TCT GTC AT-3′ and left primer 5′-CGG CAT CCG TTG TCG GTT TCA CAA ATG CCT TGC AGT G-3′ for PPAR*γ*, and right primer 5′-ACC CAC ACT GTG CCC ATC TA-3′ and left primer 5′-CGG AAC CGC TCA TTG CC-3′ for *β*-actin. The amplified RT-PCR products were analysed in 2% agarose gels, visualized by ethidium bromide staining, and photographed under ultraviolet light. 

### 2.8. Morphology Change and Apoptotic Cells 

For morphological analysis, after incubation of brazilein (5, 10, and 20 **μ**M) for 72 h, the BCC cells in each well were washed once with 1 × PBS, and analysis was performed using a phase contrast inverted light microscope (Nikon, TE2000-U, Japan). To assess specific apoptosis, cells (1 × 10^5^  cells/mL) were plated in 24-well plates and then treated with 5 **μ**M brazilein for 72 h. After incubation, cells were washed by PBS and fixed with 4% paraformaldehyde and stained with Hoechst 33342 (0.1 **μ**g/mL) at 37°C for 10 min in the dark. The nuclear morphology changes were viewed under a fluorescent microscope (Nikon, TE2000-U, Japan). 

### 2.9. Caspase-3 Expression Analysis

For western blotting, BCC cells (1 × 10^5^ cells/mL) were treated with brazilein (10 **μ**M) for 72 h. Then, cells were harvested, washed with PBS, and incubated for 20 min at 4°C in 1 mL of lysis buffer containing 50 mM Tris-HCl, pH 7.5, 1% Triton X-100, 5 mM EGTA (ethylene glycol-bis(2-aminoethylether)-*N*,*N*,*N *′,*N *′-tetraacetic acid), 150 Mm NaCl, and 1 mM phenylmethylsulfonyl fluoride (PMSF). The cell lysates containing 40 **μ**g of solubilized protein were subjected to 12% sodium dodecyl sulfate-polyacrylamide gel electrophoresis (SDS-PAGE) and electrophoretically transferred to nitrocellulose membranes. The membranes were blocked in 5% skim milk. Blots were incubated with the antibodies against cleaved Caspase-3 (Cell Signaling Technology) and *β*-actin (Santa Cruz, CA, USA). The membranes were incubated with the appropriate secondary antibody conjugated with horseradish peroxidase (Bio-Rad, Hercules, CA, USA). Blotted antibodies were visualized by chemiluminescence method (ECL kit, Amersham).

### 2.10. Preparation of *H. nana* Adult Worms

*H. nana* adult worms were obtained from each part of the intestines of wild type mice, purchased from Lin's farm in Fengshan, Kaohsiung, Taiwan. These parts of the intestine were the duodenum, jejunum, ileum, colon, and rectum. The *H. nana* had an average length of 5–50 mm and were collected using a needle with a blunt tip, before being placed in Petri dishes with 0.9% NaCl and gentamycin (10 mg/mL). They were then washed several times. The adult worms were individually observed under an inverted microscope and those that exhibited any kind of internal or external damage were discarded. The adult worms were then identified by their morphological features, divided into groups, and placed in 24-well plates (which contained cultivated media RPMI-1640 plus 20% FBS, pH 7.4, in an atmosphere of 95% O_2_/5% CO_2_, 37°C). These culture conditions have been shown to maximize the development and survival of *H. nana*.

### 2.11. Assay of Oscillation and Peristalsis Activities of *H. nana*

The above *H. nana* cultivated media were supplemented with L-glutamine (2 mM), penicillin (100 IU/mL), streptomycin (100 mg/mL), and amphotericin B (0.25 **μ**g/mL), and then the effects of brazilein at concentrations of 10, 50, and 100 **μ**M were tested. The survival and mobility of the adult worm were assessed at 2, 4, 6, 12, 24, 48, and 72 h using a stereomicroscope. The oscillation and peristalsis states of adult worms were scored blindly by two investigators. Cestode activity was scored by monitoring both oscillation and peristalsis. Oscillation was scored of movement at scolex and neck for each second for 30 second, and then the highest score was 30. Peristalsis record the contraction real times at scolex and neck. All data were compared with the initial time before the test compounds had been added. Worms death and complete standstill as determined by none any oscillation and peristalsis were appeared for 30 second (defined as death).

### 2.12. A. simplex Larvae Preparation 

The *A. simplex *third-stage larvae (AsL3) were obtained from the muscle and peritoneum of fresh *Trichiurus lepturus*(largehead hairtail, Atlantic cutlassfish) that were purchased from the fish market of Kaohsiung, Taiwan. The AsL3 had an average length of 20–22 mm and were collected using a needle with a blunt tip, placed in Petri dishes with 0.9% NaCl, and washed several times. The majority of the larvae were encysted, but they quickly became excysted upon washing in NaCl solution. They were individually observed under an inverted microscope and those that showed any internal or external damage were discarded. The larvae were then identified by morphological features, divided into groups and placed in 24-well plates (which contained cultivated media RPMI-1640 plus 20% FBS, pH 4.0, in an atmosphere of 95% O_2_/5% CO_2_, 37°C). These culture conditions have been demonstrated to provide for the maximum development and survival of *A *[19, 34]. 

### 2.13. Assay of Lethal Efficacy and Loss of Spontaneous Mobility of *A. simplex*

The above AsL3 cultivated media were supplemented with L-glutamine (2 mM), penicillin (100 IU/mL), streptomycin (100 mg/mL), and amphotericin B (0.25 **μ**g/mL) and tested of brazilein for 10, 100, and 200 **μ**M. The survival and mobility of the larvae were assessed at 2, 4, 8, 12, 24, 48, and 72 h using a stereomicroscope. Two investigators blindly scored the larvae as dead, with poor mobility or with normal mobility. The percentage losses of spontaneous motion during 3 min periods immediately after incubation and complete standstill were determined by stimulation 4-5 h later (defined as death). The nematocidal activity was modified according to a scoring system that was originally developed by Kiuchi et al. [35] and Lin et al. [19].

### 2.14. Statistical Analysis

The results are expressed as mean ± standard  deviation (SD). Statistical differences were estimated by one-way analysis of variance (ANOVA) followed by Dunnett's test or the Tukey-Kramer test. A *P* value of 0.05 was regarded as significant. The data were analyzed and the figures plotted using software (SigmaPlot Version 8.0 and SigmaStat Version 2.03, Chicago, IL, USA).

## 3. Results and Discussion

### 3.1. Antioxidant Activity of Brazilein 

The antioxidant capacity of brazilein was evaluated by performing DPPH^•^ and ABTS^•+^ tests. The scavenging of DPPH^•^-radicals is routinely performed as a preliminary test for estimating the antioxidant activity of natural compounds [36]. The DPPH^•^-radical scavenging activity indicates the ability of an antioxidant compound to donate electrons or hydrogens and thereby become a more stable molecule.  Figure 1(b) presents the effects of brazilein (5, 10, 20, 30, 40, 50, and 100 **μ**M) and trolox on the scavenging activities of DPPH^•^-radicals, determined by spectrophotometry. The ability of brazilein to scavenge DPPH^•^ radicals increased with its concentration, as does that of trolox, which is well known for its antioxidant properties. The EC_50_ values of brazilein for the scavenging of DPPH^•^-radicals were 27.6 **μ**M (brazilein) and 20.1 **μ**M (trolox). 

The radical monocation of ABTS^•+^ is carried out by the oxidation of ABTS using potassium persulfate and is reduced in the presence of the hydrogen-donating antioxidants [30].  Figure 1(c) displays the spectrophotometrically determined activities of brazilein (5, 10, 20, 30, 40, 50, and 100 **μ**M) and trolox in scavenging ABTS^•+^-radicals. The EC_50_ values for the scavenging of ABTS^•+^-radicals were 26.5 **μ**M (brazilein) and 25.0 **μ**M (trolox). The TEAC method is based on the capacity of various substances to scavenge the ABTS^•+^-radical cations and the comparison of those capacities with that of a standard antioxidant (trolox), using a dose-response curve. The antioxidant capacity of brazilein that was measured as a TEAC value, was 981.1 mg of trolox/g. 

Antioxidant activity can be evaluated by measuring the ability of a substance to reduce the damage that is caused by free radicals. Some mechanisms that are involved in many human diseases, such as hepatocarcinogenesis, diabetes, malaria, acute myocardial infarction, and skin cancer involve lipid peroxidation as a main source of membrane damage [37]. To examine the potential mechanisms by which brazilein prevents oxidative damage, the inhibition of lipid peroxidation by brazilein was investigated by measuring the malonaldehyde content.  Figure 1(d) plots the concentration-dependent inhibition of lipid peroxidation by brazilein (5, 10, 20, 30, 40, 50, and 100 **μ**M). The EC_50_ values of the inhibition of lipid peroxidation efficiency by brazilein, trolox, and rutin were 14.5, 14.9, and 5.1 **μ**M, respectively. Although the inhibition of lipid peroxidation activity by brazilein was weaker than that by rutin, brazilein and trolox had similar effects on lipid peroxidation. 

Brazilein contains hydroxyl in phenol and hydroxyl functional groups, which may be responsible for its ability to scavenge free radicals and inhibit the formation of lipid peroxyl radicals.

### 3.2. Brazilein Suppresses Intracellular Lipid Accumulation during Adipocyte Differentiation

**Figure 2:**
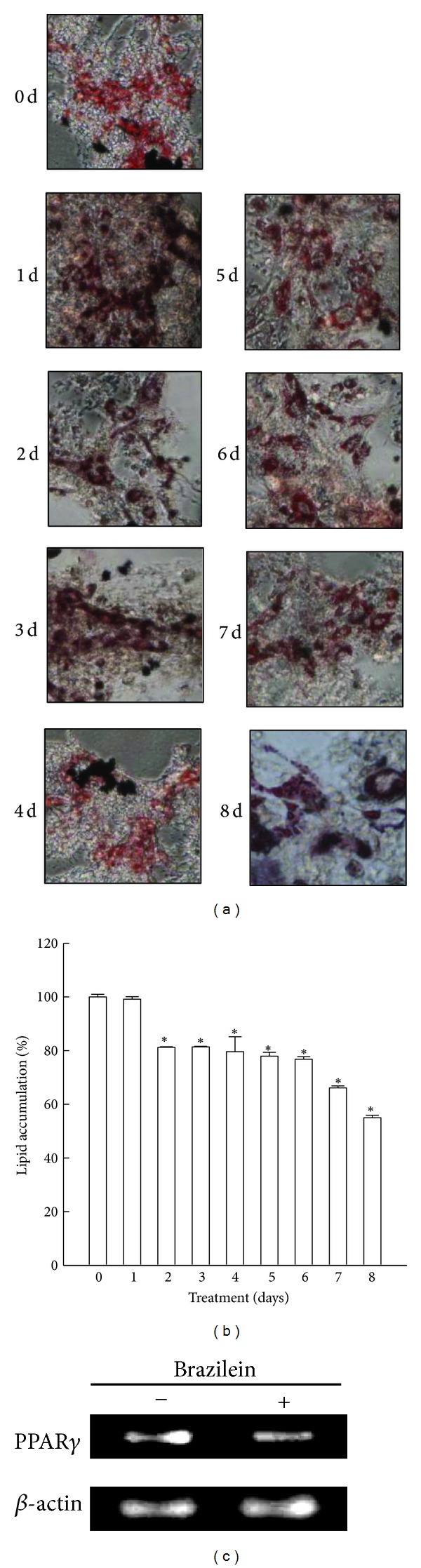
Inhibition of accumulation of lipid droplets by brazilein in 3T3-L1 cells. (a) Differentiated 3T3-L1 cells were treated with brazilein (5 **μ**M) for eight days. Lipid accumulation was measured by Oil Red O staining. Photomicrographs present Oil Red O staining of differentiated cells that were treated with brazilein. (b) Percentage of lipid accumulation was analyzed by quantitative analysis of Oil Red O staining. Data are presented as mean ± SD from three independent experiments; *P < 0.05 indicates significant difference from vehicle-treated cells. (c) The gene expressions of PPAR*γ* and *β*-actin were determined by RT-PCR.

Increased body weight has been reported to result in increased oxidative stress [38]. Numerous studies have found elevated oxidative stress biomarkers in obesity and have suggested that oxidative stress may be the linking mechanism in the pathway leading from obesity to obesity-related diseases [39]. Furthermore, obesity has been shown to be associated with reduced antioxidant defense mechanisms. The differentiation of murine 3T3-L1 fibroblasts into adipocytes is one of the most dramatic and sensitive morphological alterations in cultured mammalian cells. This cell model can be utilized to screen bioactive molecules using adipogenesis profiles [40]. Excess adipogenesis caused abnormal adipokine secretion, which was accompanied by changes in the endocrinological effects of adipocytes on adipogenesis, inflammation, and energy metabolism [41]. Brazilein has been found to exhibit anti-oxidation and anti-inflammatory activities [25], and we hypothesize that brazilein may inhibit the accumulation of lipids. To quantify the effect of brazilein on adipocyte differentiation, 3T3-L1 cells that were undergoing MDI-induced differentiation were treated with brazilein (5 **μ**M). Following differentiation, the cells were fixed and stained using Oil Red O to examine neutral fat deposition. As displayed in Figure 2(a), brazilein reduced the accumulation of lipid droplets within cells below that in MDI-treated positive control cells. This result was further supported by quantitative spectrophotometric analysis of cellular neutral lipid content. As presented in Figure 2(b), the level of lipid accumulation over eight days was 54.9% of that of the MDI-treated positive control cells (100%). Adipogenesis is a differentiation process by which undifferentiated preadipocytes are converted to fully differentiated adipocytes, regulated by a highly organized cascade of transcription factors such as members of PPAR*γ* [7]. To investigate whether the reduced lipid accumulation in brazilein-treated cells was due to the inhibition of adipocyte differentiation, we examined the expression of adipogenic markers in 3T3-L1 cells. As shown in Figure 2(c), addition of brazilein (5 **μ**M) suppressed the expression of PPAR*γ* significantly as revealed by RT-PCR. In the present study, brazilein exhibited antioxidative properties and may inhibit lipid accumulation, and have antiobesity effects. 

### 3.3. Effect of Brazilein on Cell Viability and Skin Cancer Cell Apoptosis

**Figure 3:**
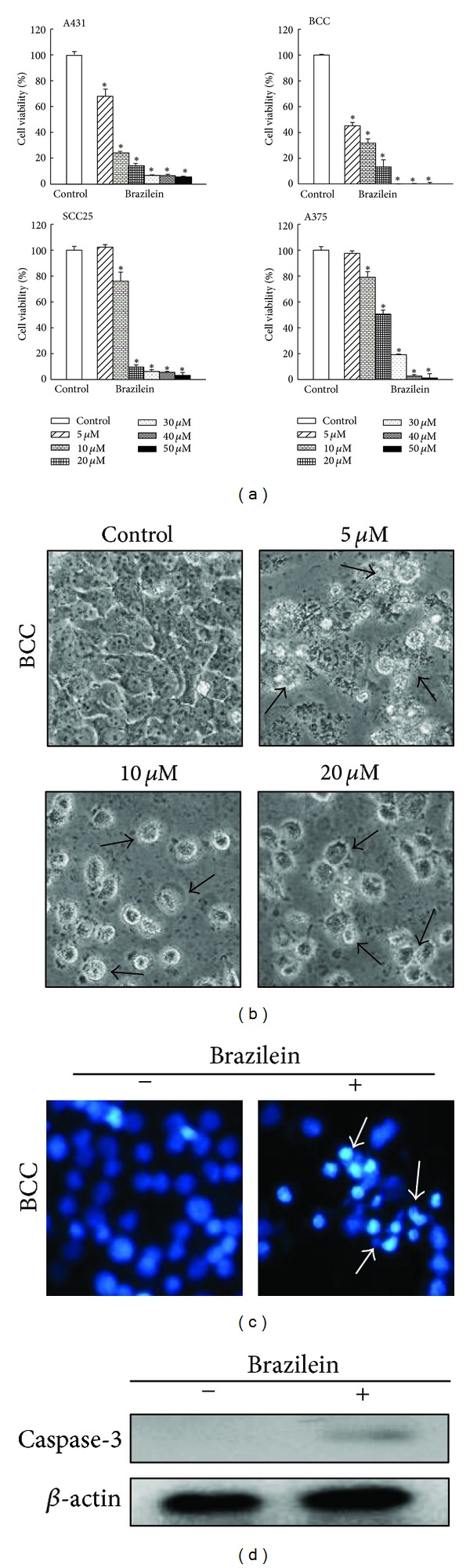
Effect of brazilein on cell viability in skin cancer cells. (a) Cell viability of brazilein (5, 10, 20, 30, 40, and 50 **μ**M) to skin cancer cells (A431, BCC, SCC25, and A375) for 72 h and assessed by MTT assay. Each value is presented as mean ± SD of three individual experiments; *P < 0.05 indicates a significant difference from vehicle-treated cells. (b) Morphological changes induced by brazilein in BCC cells. BCC cells were treated with brazilein (5, 10 and 20 **μ**M) and without (control) for 72 h. Apoptotic cells (arrows) were characterized by cellular shrinkage and rounded cell bodies (phase-contrast-inverted microscopic, 200x). (c) Nuclear morphological changes caused by brazilein in BCC cells. BCC cells were treated with (+) or without (−) brazilein (5 **μ**M) for 72 h, and then the nuclear was stained with Hoechst 33342. Under a fluorescent microscope, apoptotic cells (arrows) were characterized by marked nuclear condensation, shrinking, and fragmentation (200x). (d) Activation of caspase-3 induced by brazilein. BCC cells were treated with (+) or without (−) brazilein (10 **μ**M) for 72 h, and the caspase-3 and  *β*-actin expressions were determined by western blot.

The concept that consumption of antioxidant rich foods may prevent cancer or improve treatment has been supported by some studies. Results of these studies indicate that vitamins and certain phytochemical antioxidants including flavonoids and carotenoids, are effective against the proliferation of various cancer cells [42]. Brazilein reportedly inhibits the growth of several cancer cells, including liver, breast, lung, and gingival cancer cells [27]. The IC_50_ values of brazilein were 11.3 and 12.2 **μ**M for human liver cancer HepG2 and Hep3B cells, 8.4 and 14.1 **μ**M for breast cancer MDA-MB-231 and MCF-7 cells, 34.6 **μ**M for lung cancer A549 cells, and 30.8 **μ**M for gingival cancer Ca9-22 cells [27]. However, the antitumor effect of brazilein on human skin cancer cells is not fully understood. In this study, the cytotoxic activity of brazilein against human skin cancer cells (epidermoid carcinoma A431 cells, basal cell carcinoma BCC cells, squamous cell carcinoma SCC25 cells, and malignant melanoma A375 cells) was investigated by MTT assay, and relevant results are plotted in Figure 3(a). After 72 h of treatment, brazilein (5, 10, 20, 30, 40, and 50 **μ**M) had significant antiskin cancer effects against A431, BCC, SCC25, and A375 cancer cells by affecting cell proliferation; the inhibitory effect was dose-dependent. The IC_50_ values of brazilein against skin cancer cells were 7.0 **μ**M (A431), 4.5 **μ**M (BCC), 13.0 **μ**M (SCC25), and 20.2 **μ**M (A375). 

**Figure 4:**
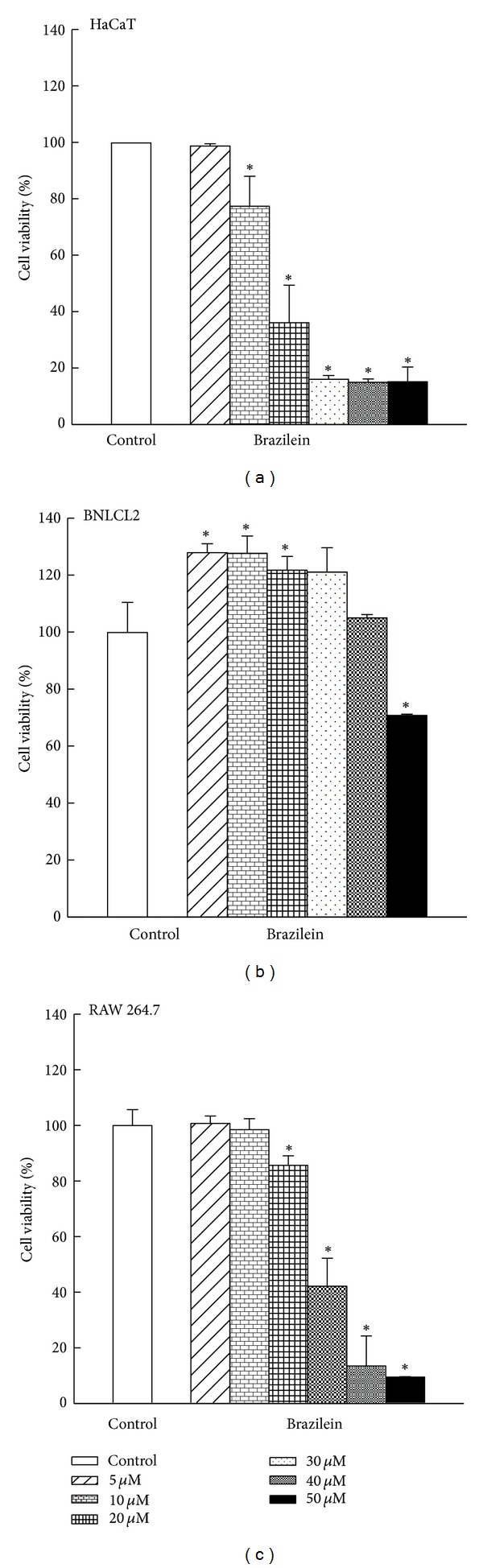
Effect of brazilein on cell viability in noncancerous cells (HaCaT and BNLCL2) and mouse leukemic monocyte macrophage RAW 264.7 cells. Cells were treated with brazilein (5, 10, 20, 30, 40, and 50 **μ**M) for 72 h. Cell viability was evaluated with MTT assay. Each value is presented as mean ± SD of three individual experiments; *P < 0.05 indicates a significant difference from vehicle-treated cells.

The morphological changes in skin cancer cells that had been treated with brazilein were studied to determine whether a particular mechanism of death was responsible for the decrease in viability of those cells. As displayed in Figure 3(b), under phase-contrast-inverted microscopic examination, the treatment of BCC cells with brazilein (5, 10, and 20 **μ**M) for 72 h caused the cells to lose their shape, the cell bodies to become rounded, and the cells subsequently to detach from the surface. After the BCC cells had been treated with brazilein (5 **μ**M) for 72 h, nuclear condensation, shrinking, and fragmentation were identified by Hoechst 33342 staining under a fluorescent microscope (Figure 4(c)). Apoptosis is characterized by cell shrinkage, DNA fragmentation, membrane blebbing, and apoptotic bodies [31]. The experimental data herein suggest that brazilein-treated BCC cells die and exhibit the morphological features of apoptosis. 

Caspases, a family of cysteine proteases, are expressed in almost all types of cell as inactive proenzymes. Extensive evidence suggests that the activation of caspase triggers the apoptotic process in various cells. Caspase-3 has a pivotal role in the terminal phase of apoptosis [43]. To determine whether caspases are involved in the brazilein-induced apoptosis of skin cancer cells, the caspase-3 expression of BCC cells was investigated by western blotting following the exposure of those cells to brazilein (10 **μ**M) for 72 h (Figure 3(d)). The experimental results thus obtained suggest that brazilein induces cell apoptosis via caspase-dependent cell death pathways in skin cancer cells.

The cytotoxic effects of brazilein on noncancerous cells (human premalignant keratinocytic HaCaT cells and mouse embryonic liver BNLCL2 cells) and mouse leukemic monocyte macrophage RAW 264.7 cells were examined by MTT assay. As shown in Figure 3, treatment with brazilein for 72 h resulted in a dose-dependent reduction in HaCaT and RAW 26.47 cell viability. However, the inhibitory effect of brazilein in BNLCL2 cell viability was largely refractory. The IC_50_ values of brazilein against HaCaT, BNLCL2, and RAW 264.7 cells were 17.7 **μ**M, >50 *Μ*m, and 28.2 **μ**M, respectively. These experimental results suggest that the inhibition of cell growth by brazilein is greater for skin cancer cells (A431, BCC, and SCC25) than for A375, RAW 264.7, and noncancerous cells (HaCaT and BNLCL2).

### 3.4. Cestocidal Activity against *H. nana*

**Figure 5:**
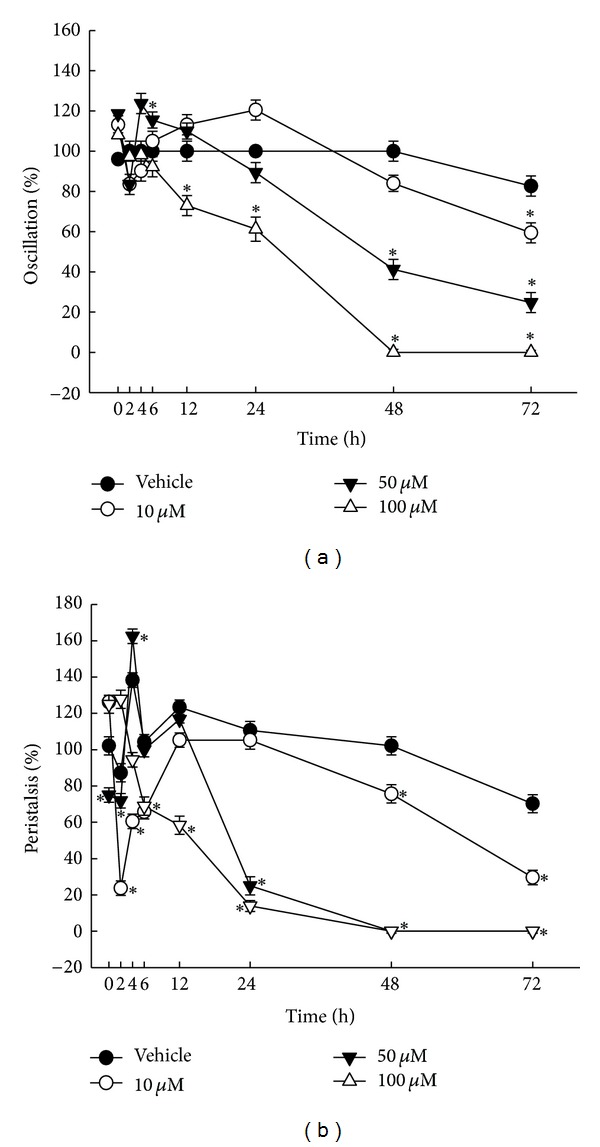
Effect of brazilein on *H. nana*. Treatment with various concentrations of brazilein (10, 50 and 100 **μ**M) with incubation times of 2, 4, 6, 12, 24, 48, and 72 h on *H. nana*, respectively. Time course of effect on oscillation (A) and peristalsis (B) of *H. nana* of brazilein presented as percentages. Vehicle is 0.1% DMSO solvent. Each value is presented as mean ± SD of three individual experiments; *P < 0.05 indicates a significant difference from the result for vehicle-treated worms.

Figures 5(a) and 5(b) plot the time course of oscillation and peristalsis during brazilein treatment. In oscillation activity assay, vehicle control (0.1% DMSO) was decreased 17% from 72 h cultivation (Figure 5(a)). However, in the peristalsis activity assay, vehicle control (0.1% DMSO) was decreased 30% from 72 h cultivation (Figure 5(b)). The change of peristalsis of *H. nana* was more sensitive than did oscillation by treatment of vehicle. Treatment with 10, 50, and 100 **μ**M brazilein has a greater effect of peristalsis than did oscillation for 24, 48, and 72 h. Peristalsis activity disappeared before oscillation activity was lost when *H. nana* was dead. In fact, *H. nana *has no peristalsis or oscillation effect when it is dead. 

In the oscillation activity assay (Figure 5(a)), exposure to 100 **µ**M brazilein for 48 h caused the death of 100% of *H. nana*. Treatment with brazilein (a concentration of 50 or 100 **µ**M but not 10 **µ**M) for 48 and 72 h reduced the oscillation up to 60% and 100%, respectively. Brazilein at a concentration of 100 **µ**M slowly reduced oscillation from 2 to 72 h. Other concentrations of brazilein reduced the oscillation activity but did not cause death. Brazilein reduced the oscillation activity of *H. nana* in a time- and dose-dependent manner for 24 to 72 h (Figure 5(a)). 

The effect of brazilein over time of the peristalsis activity of *H. nana *was investigated (Figure 5(b)). In general, in peristalsis activity assay, a dose- and time-dependent effect for 24 to 72 h was also observed by treatment with brazilein. Treatment for 24 h with 50 and 100 **µ**M brazilein stopped peristalsis in more than approximately 75% of worms. Brazilein at 100 **µ**M slowly reduced peristalsis from 2 to 72 h. It killed all worms. Brazilein at a concentration of 50 and 100 **µ**M stopped peristalsis in 48 h. Treatment with 10 **µ**M brazilein for 72 h reduced peristalsis to 29%. This effect on peristalsis is stronger than that on oscillation activity. 

### 3.5. Larvicidal Activity against *A. simplex* and Elimination of Its Spontaneous Activity 

**Figure 6:**
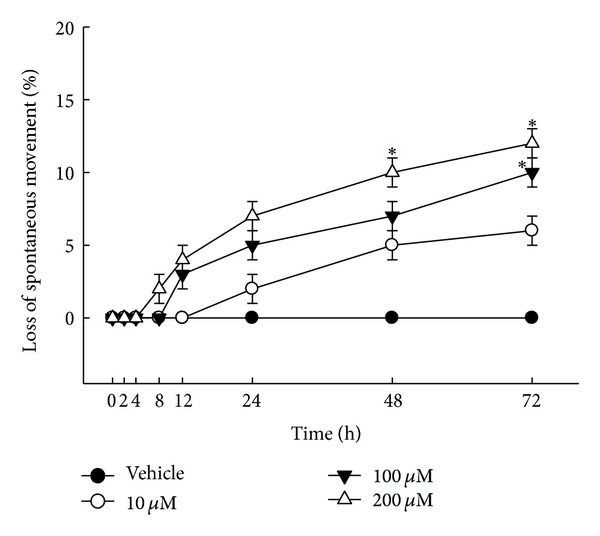
Time course of elimination of spontaneous movement of *A. simplex* by brazilein treatment. Effect of brazilein (10, 100, and 200 **μ**M) for 2, 4, 8, 12, 24, 48, and 72 h on third-stage larvae of *A. simplex* (AsL3). Vehicle is 0.1% DMSO solvent. Each value is presented as mean ± SD of three individual experiments. Statistically significant *P < 0.05 indicates a significant difference from the result for vehicle-treated worms.

In the first series of experiments, the larvicidal effects were used to study the ability of brazilein to alter survival of AsL3. Treatment with 10, 100, or 200 **μ**M brazilein for 24–72 h did not measurably affect larvae survival (data not shown). The time course of the brazilein-induced loss of mobility on AsL3 was also studied. As shown in Figure 6, more than 5% of the worms had stopped moving at 48 h of treatment with 10, 100, and 200 **μ**M brazilein, whereas up to 10% of the larvae had ceased movement activity at 72 h of treatment with 200 **μ**M (Figure 6). Additionally, the maximum loss of spontaneous movement occurred at a concentration of 200 **μ**M for 72 h. Generally, brazilein caused a dose- and time-dependent loss of spontaneous movement. However, the vehicle (0.1% DMSO) had no effect on AsL3. 

Free radical scavenging activities have been involved in some pathological conditions such as inflammatory diseases, cancer, and aging [44]. Some agents have been found to have antibacterial and antiprotozoal activities, at least partially, by means of an antioxidant property, though they were not sure to what extent this affected anthelminthic activity [45–47]. Otherwise, free radical scavenging may reduce anthelminthic activity by permitting larvae survival [48]. 

Therefore, it is still not clear how free radical savaging may affect worms survival of certain anthelminthic agents. In this study, we first identified brazilein has antiparasitic activities on AsL3 of *A. simplex *and adult worms of* H. nana in vitro*, and then used DPPH and ABTS^•+^ scavenging assay and inhibition of lipid peroxidation assay to determine whether or not there was an association between the possible scavenger activity of brazilein and its anthelminthic activity against *A. simplex *and *H. nana*.

This study is the first to determine the anthelminthic activity of brazilein on *A. simplex *and *H. nana. *Thus we found that brazilein has not only anthelminthic activity for L3 of *A. simplex *and adult worm*s of H. nana* but also radical scavenging activity against DPPH and ABTS^•+^ and inhibit the formation of lipid peroxyl radicals. So brazilein with radical scavenging activity has not reduced its anthelminthic activity but further investigations for the mode of brazilein actions or/and mechanisms for its anthelminthic effects between free radical scavenging activity and inhibition of lipid peroxidation relationship are necessary. These results might be useful in the search of more selective and efficient naturally anthelminthic compounds.

## 4. Conclusion 

These results reveal that brazilein, a purified substance from *Caesalpinia sappan* L., scavenges free radicals and inhibits lipid peroxidation, indicating antioxidation activity. This is the first study in which brazilein is found to suppress the differentiation of 3T3-L1 preadipocytes to adipocytes. Treatment with brazilein reduced the intracellular accumulation of neutral lipids and suppressed the induction of PPAR*γ*. Furthermore, this study presented evidence that skin cancer cells are sensitive to brazilein-induced cytotoxic effects, which are mediated by the induction of apoptosis via a caspase-3-dependent pathway. Additionally, studies have shown that brazilein has a stronger anthelminthic effect on *H. nana *than on *A. simplex.* These results support the development of selective and efficient natural anthelminthic compounds against helmineth or cestode. In the DPPH^•^ and ABTS^•+^ scavenging assays, the ability of brazilein to scavenge radicals was evaluated. Previous evidence has established that larvicide activity toward *A. simplex* dose not depend on scavenging activity and also that free radicals can be harmful to *A. simplex*, in which case the scavenging of these free radicals permits larvae to survive. However, this study is the first that brazilein eliminates the spontaneous movement of AsL3, has cestocidal effects against *H. nana* exhibits scavenging activity against DPPH^•^ and ABTS^•+^ radicals, and inhibits the formation of lipid peroxyl radicals. Therefore, the radical scavenging activity of brazilein does not reduce its ability to stop the spontaneous movement of AsL3 and or its cestocidal effects on *H. nana*. Further investigations must be conducted to elucidate the anthelminthic mechanisms of brazilein against *A*. *simplex* and *H. nana* and its ability to eliminate the spontaneous movement of *A*. *simplex* and *H. nana* and their relationships to free radical scavenging activity.
